# Role of Multidetector Computed Tomography Urography in the Evaluation of Obstructive Uropathy: A Review

**DOI:** 10.7759/cureus.48038

**Published:** 2023-10-31

**Authors:** Shreya Khandelwal, Rajasbala Dhande, Anshul Sood, Pratapsingh Parihar, Gaurav V Mishra

**Affiliations:** 1 Radiodiagnosis, Jawaharlal Nehru Medical College, Datta Meghe Institute of Higher Education and Research, Wardha, IND

**Keywords:** multidetector computed tomography (mdct), obstructive uropathy, urinary bladder, renal calculi, pelvi-ureteric junction obstruction

## Abstract

Obstructive uropathy, a prevalent clinical problem, can irreparably harm the kidneys if not treated promptly. As a result, accurate diagnosis is necessary for prompt management. This study examines the utility of multidetector computed tomography (MDCT) urography in identifying obstructive uropathy. PubMed, Google, Embase, Medline, and other electronic databases were used to search the English-language literature. The search phrases were obstructive urinary infections or urinary bladder or kidneys or MDCT. The authors’ expertise and experience in the subject area aided in archiving pertinent publications. Even though the dilated upper tract of the ureters can be seen, ultrasonography (USG) has limitations because it cannot show the middle portion of the ureters, even if they are dilated mostly due to bowel gas artifacts. The USG does not emphasize the functioning of the renal tract. To evaluate obstructive uropathy, MDCT urography plays a very important role. For speedy, effective therapy, it provides a quick diagnosis of the source of obstruction.

## Introduction and background

Blockages in the regular urine flow, which may be caused by anatomical or functional abnormalities of the urinary system, are the hallmarks of obstructive uropathy [1]. Obstruction can occur anywhere throughout the urinary tract, including the renal tubules, renal pelvis, ureter, bladder, and urethra. Several hereditary and acquired diseases can cause extraluminal or intraluminal urinary obstruction. Urinary tract blockage can occur at any stage of life, including throughout fetal development. Blood clots, scarring, stones, papillae sloughing, and other intraluminal diseases can all result in urinary system blockages. An enlarged uterus, trauma, a cancer stricture, and swollen lymph nodes that impinge on the ureter cause obstruction are examples of extraluminal causes. Depending on where it is, it may cause a unilateral or bilateral obstruction [2].

There is evidence that the symptoms of obstructive uropathy usually include groin and/or stomach discomfort, nausea, vomiting, profuse perspiration, or diaphoresis [3]. Although there are several underlying causes of obstructive uropathy, studies show that kidney, ureter, and urinary tract stones are the most typical ones [4]. Other medical conditions, including pregnancy, prostate cancer, retroperitoneal fibrosis, spinal cord injury, ureteral stricture, and congenital anomalies like ureteropelvic junction obstruction, may also contribute to obstruction and stones [5-7]. How efficiently urine is transported is determined by the development of connections between the morphology and physiology of the organs, particularly the kidneys and the ureters. When there is kidney blockage, the proliferation of smooth muscle cells lining the pelvis ureter is more prone to harm [1].

Damage to the renal pelvis and poor smooth muscle differentiation are the leading causes of physiological obstruction in the urinary system. Several imaging modalities, including intravenous urography, radiography, ultrasound, computed tomography (CT), magnetic resonance imaging (MRI), and radionuclide tests, are available to evaluate patients with obstructive uropathy. Ultrasonography (USG) beats intravenous urography (IVU) in obstruction circumstances in detecting the collecting system dilatation. USG has limits since it cannot show the middle part of the ureters, even if they are dilated, although the dilated proximal urinary tract is apparent [1]. Additionally, USG does not reveal the renal tract’s functioning status [8]. Despite being a relatively efficient imaging technique for obstructed urinary systems, MR urography provides less accurate diagnostic picture quality than CT urography [8].

MR urography is also expensive and time-consuming [9]. Urinary tract imaging has advanced beyond previously conceivable thanks to modern CT technologies. The capacity to obtain virtual cystoscopy images and rapid capture with enhanced temporal-spatial resolution are all features of new multidetector CT (MDCT) scanners [10]. For many urological conditions, such as urolithiasis, obstructive uropathy, urinary tract infections (UTI), renal malignancies, and trauma, CT is increasingly the preferred examination. The architecture of the urinary system can be precisely viewed with CT urography [9]. It also makes any extrinsic urinary blockage very clear to see. As a result, the current review was conducted to evaluate the contribution of MDCT to the assessment of obstructive urography.

## Review

Materials and methods

The following goals are being pursued with this review: the role of MDCT in obstructive urography.

PubMed, Google, Embase, Medline, and other electronic databases were used to search the English-language literature. The search phrases were obstructive urinary infections or urinary bladder or kidneys and MDCT. The writers’ familiarity with the field and knowledge of the resources available greatly facilitated the compilation of this database. The articles in this review meet the following requirements: 1. Included are studies in English. 2. Studies from the last 10 years are also included. 3. Some studies are solely about urinary blockages MDCT.

Study Selection and Eligibility Criteria

Studies involving obstructive urinary infections, MDCT, and urinary tract obstruction that were published in English-language publications till 2023, were considered. Included studies also described the outcome in terms of radiological data. Case reports, case series, research involving studies on animals, reviews, and articles with insufficient data were excluded from the list. Any studies including studies other than MDCT were excluded.

Data Extraction and Synthesis

Researches were selected considering three different processes. A careful examination of the research’s title was done to include the necessary research that met the inclusion and exclusion criteria. Abstracts were gathered and assessed. Studies that meet the eligibility criteria were included after reading the complete text. The investigation finally focused on four items, as displayed in Table 1.

**Table 1:** Data extraction and synthesis

Initial search	100
Duplicates and non-relevant	41
Case reports and series	13
Reviews	31
Abstract	05
Language other than English	06

Results

**Figure 1:**
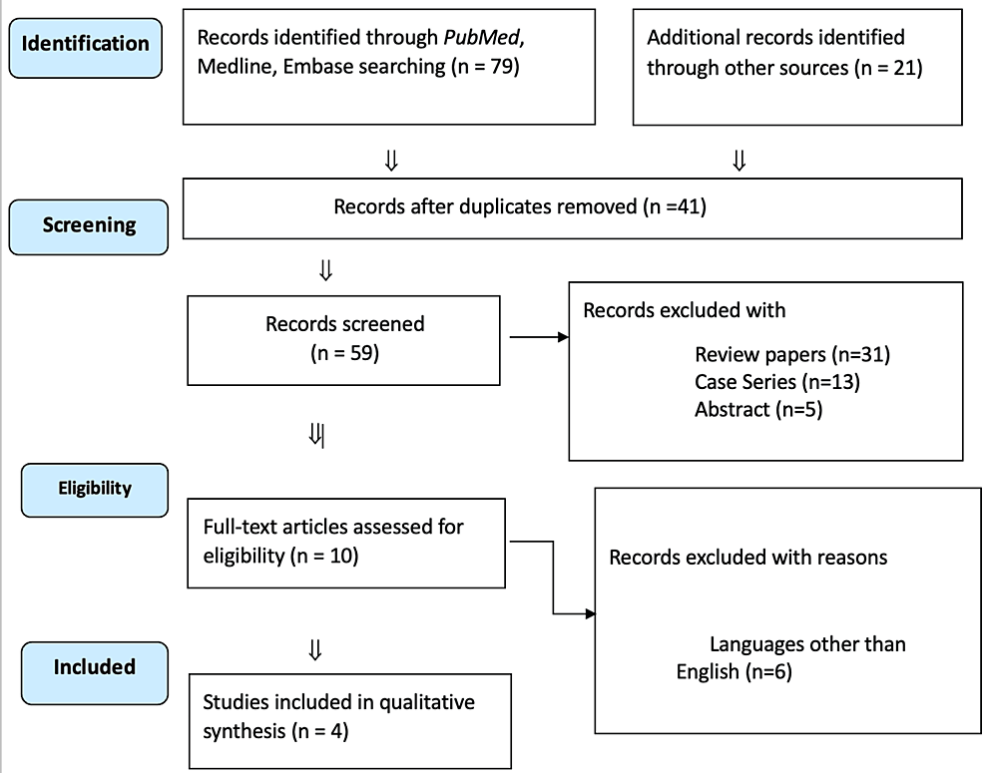
PRISMA Flowchart The flowchart was created by the authors of this article.

The initial search consisted of 100 articles. After the removal of duplicates, articles not meeting the eligibility criteria were removed and four papers were considered for analysis (Figure 1).

The studies’ quality was evaluated using the Cochrane collaborative approach for assessing the risk of bias in RCTs. The “Newcastle-Ottawa Quality Assessment Form” was used to establish the accuracy of the analysis of the data for this systematic review [11-13]. Modified Jadad scoring system has been used to assess the bias and blinding outcomes and research findings have been presented in brief in tabular form (Table 2) [14]. Moreover, the extracted data is shown in Table 3.

**Table 2:** Assessment of quality of studies included in present systematic review

Authors name	Selection bias	Allocation concealment	Reporting bias	Others	Performance bias blinding participants and personnel	Blinding outcome	Attrition bias
Soomro et al. in 2016 [15]	Low risk	Low risk	Low risk	Low risk	Low risk	Unclear	High risk
Park et al. in 2016 [16]	Low risk	Low risk	Low risk	Low risk	Low risk	Low risk	Low risk
Sudah et al. in 2016 [17]	Low risk	Low risk	Low risk	Low risk	Low risk	Low risk	Low risk
Sharma et al. in 2018 [18]	Low risk	Unclear	Low risk	Low risk	Low risk	Unclear	Low risk

**Table 3:** Population data table MDCT: multidetector computed tomography; SWL: shock wave lithotripsy; MRU: magnetic resonance urography

Author and year	Patients	Intervention	Outcomes
Soomro et al. in 2016 [15]	60 patients of kidney stone	64-slice MDCT	The stone size measured using the soft-tissue window setting on an MDCT is significantly different from the measurement on the bone window setting.
Park et al. in 2016 [16]	223 patients with single proximal ureteral stones	64-channel unenhanced helical CT system	Patient and CT-based parameters—including BMI, stone diameter, and perinephric edema—are independent predictors of the outcome of SWL of proximal ureteral stones.
Sudha et al. in 2016 [17]	20 patients (39 UUT excreting units)	3.0T- magnetic resonance urography (MRU) protocol versus triple-phase computed tomography urography (CTU)	Comprehensive 3.0T-MRU is an accurate imaging modality achieving comparable performance with CTU; since it does not entail exposure to radiation, it has the potential to become the primary investigation technique in selected patients.
Sharma et al. in 2018 [18]	50 consecutive subjects presenting with evidence of unilateral or bilateral hydronephrosis	MDCT	MDCT urography is very useful for complete evaluation of obstructive uropathy and allows rapid detection of the level and cause of obstruction which is critical for timely and effective management.

Discussion

MDCT urography, which permits fast imaging simultaneous evaluation of intraluminal extraluminal causes and site of obstruction, along with a comment on the status of functioning of the kidney, is a promising imaging approach for obstructive uropathy. Subjects with bilateral obstructive uropathy were excluded from the case study, highlighting that bilateral blockage is less frequent than unilateral obstruction.

The ureter and the renal pelvis, specifically the vesio-ureteric junction, can develop calculi, consistent with several other research [19-21]. Non-contrast computed tomography (NCCT), which can precisely identify the position size of the calculus and related changes in back pressure, is the best imaging approach for finding urinary calculi [22]. Concurrently with MDCT urography, the kidneys’ level of functioning can be evaluated [20]. Urinary bladder masses are the second most common cause of urinary tract obstruction owing to the involvement of the vesicoureteric junction (VUJ). In our study, bilateral and unilateral VUJ involvement was involved in two and six subjects, respectively. It is comparable to the study conducted by Moawad et al. [20]. Multifocal transitional cell carcinomas are the most typical in the urinary bladder. With MDCT urography, rapid localization lesion count identification is achievable. Additionally, it enables the simultaneous detection of transmural tumor growth, significant lymphadenopathy, and any associated metastases [23]. Other less common causes of obstructive uropathy included ureteric stricture, PUJ obstruction, and ureteric constriction by enlarged lymph nodes.

Additionally, the discovery of other significant associated findings, other than obstructive uropathy by MDCT can significantly impact patient care. In studies across the literature, ureteric calculi have been reported to be the leading cause of the obstruction of the urinary tract system. In many of these cases, patients report having acute obstructive symptoms. An early, accurate diagnosis is essential for both short- and long-term consequences. The most important diagnostic method for evaluating urinary tract calculi is MDCT, which replaces excretory urography [24,25]. There are numerous causes for these. With MDCT, accurate isotropic 3-dimensional images can be captured, unlike excretory urography, which may involve bowel preparation and is vulnerable to artifacts from overlapping structures. Because structures do not overlap as they do in radiography, it is feasible to define even minute calculi clearly. The treatment of the urinary tract calculus depends on the size of the calculus and is best appreciated on CT. Direct multiplanar reconstructions are another feature of MDCT that allows for 3-dimensional stone renderings even more accurate measurements [15,26,27].

MDCT also provides precise representations of the weight and distance from the skin surface of the calculus. Some evaluation of the composition of the calculus can be done by using the Hounsfield units (HU). The density of the calculus helps in comparing the role of shock wave lithotripsy (SWL) and percutaneous nephrolithotomy or ureteroscopy [28,29]. MDCT urography is an adjuvant to cystoscopy because of its efficient evaluation of the entire urinary tract’s transitional carcinomas. [16,30-32]. Additionally, it enables the concurrent assessment of several lesions and any concurrently enlarged lymph nodes. Few recent studies using 3 Tesla MR urography with contrast show comparable results to MDCT urography in terms of evaluation of the upper urinary tract and is beneficial to the patient as it prevents potential radiation exposure. However, more extensive studies still need to confirm these results [17,33]. Certain disadvantages of MR urography in comparison to MDCT include increased duration of time for the scan, various artifacts, and prolonged patient stability; although these may be overcome in emergencies [34]. Up to that date, MDCT urography is still considered the gold standard.

The results of this study demonstrate that MDCT urography is one of the most valuable tests for obstructive uropathy. As MDCT urography is a multiplanar imaging modality with good spatial resolution, it helps to locate the exact site and source of obstruction. Furthermore, CT urography is superior to MR urography in deciding the treatment plan for the patient because of its efficacy in detecting the functioning of the kidney. Another benefit of MDCT is its importance in detecting metastatic lesions in the abdomen which plays an important role in determining the prognosis.

There are a few limitations of this study which includes some compromise in the bias and blinding while selecting subjects in the chosen articles. Meta-analysis is more advantageous in providing higher accuracy about the outcome of such studies.

## Conclusions

In conclusion, MDCT urography offers a comprehensive assessment of the cause and site of obstruction in the urinary tract. It may be performed quickly even with unstable patients and offers multiplanar imaging capability coupled with good spatial and contrast resolution making it the imaging modality of choice. Other associated pathologies, metastatic lesions, and abnormalities involving other abdominal organs can be easily detected by the use of high-resolution imaging. Almost all of the pathologies affecting the urinary system causing obstructive uropathy can be detected by MDCT. It is simple to add excretory phase images in patients with obstructive uropathy into CT abdominal protocols in order to get more functional information.
